# Author Correction: Brain milieu induces early microglial maturation through the BAX-Notch axis

**DOI:** 10.1038/s41467-025-58603-x

**Published:** 2025-04-07

**Authors:** Fangying Zhao, Jiangyong He, Jun Tang, Nianfei Cui, Yanyan Shi, Zhifan Li, Shengnan Liu, Yazhou Wang, Ming Ma, Congjian Zhao, Lingfei Luo, Li Li

**Affiliations:** 1 Institute of Developmental Biology and Regenerative Medicine, Key Laboratory of Freshwater Fish Reproduction and Development, Ministry of Education, Southwest University, 400715 Chongqing, P.R. China; 2 Research Center of Stem Cells and Ageing, Chongqing Institute of Green and Intelligent Technology, Chinese Academy of Sciences, 400714 Chongqing, P.R. China; 3 Department of Neurobiology and Institute of Neurosciences, School of Basic Medicine, Fourth Military Medical University, 169 Chang Le Xi Road, 710032 Xi’an, Shaanxi P.R. China; 4 Chongqing Engineering Research Center of Medical Electronics and Information Technology, School of Bioinformatics, Chongqing University of Posts and Telecommunications, 400065 Chongqing, P.R. China

**Keywords:** Differentiation, Calcium signalling, Development, Microglia, Immunogenetics

Correction to: *Nature Communications* 10.1038/s41467-022-33836-2, published online 17 October 2022

In the version of the article initially published, due to mistakes during figure preparation, the Fig. 4h (sib; neutral red image) and Supplementary Fig. S1e (neutral red, middle image; WT, 3 dpf) were inadvertent duplicates of the Fig. 2i (*bax*^*cq55*^/*Tg(NBT:bax)*; sib; neutral red). Figure 4 and Supplementary Fig. S1 have been replaced in the HTML and PDF versions of the article.

## Supplementary information

Updated Supplementary Information

