# Supplementary material for: Brain milieu induces early microglial maturation through the BAX-Notch axis
Source: Nat Commun. 2022 Oct 17;13:6117. doi: 10.1038/s41467-022-33836-2 (PMC9576735; doi:10.1038/s41467-022-33836-2)
Supplement: Supplementary file 3 — Description of Additional Supplementary Files [file 41467_2022_33836_MOESM3_ESM.pdf]

Description of Additional Supplementary Files

Supplementary Movie 1. The distribution, morphology and movement behaviours of coro1a-Dsred^{+} (c-D^{+}) cells in the WT and *bax^{cq55}* midbrains. The c-D^{+} cells are detected in WT and *bax^{cq55}* larval brain. 2 dpf WT c-D^{+} cells (PM, asterisks) are morphologically long-and-thin and they move randomly and rapidly, even exiting the observation filed. 3 dpf WT c-D^{+} cells (AM, arrows) present amoeboid morphologies and they are inert and stay locally. The c-D^{+} cells in 3 dpf *bax^{cq55}* brain (arrowheads) are morphologically and behaviourally similar to those of 2 dpf counterparts. Time-lapse images are captured for 145 min (1 frame/5 min) and single frame is generated as projections of z stacks of 16 planes with 5 μm distance, covering approximately 80 μm. The *Tg(coro1a:DsRed)* transgenic line is used.

Supplementary Movie 2. The appearance and increase of apoeb-GFP signal in an amoeboid c-D^{+} cell. A c-D^{+} cell performs phagocytosis (arrowheads) and changes to the amoeboid appearance. It then gradually turns on the expression of apoeb-GFP signal. The double transgenic line *Tg(coro1a:DsRed; apoeb:GFP)* is used. The imaging is started from 55 hpf and lasts 425 min (1 frame/5 min).

Supplementary Movie 3. Movement behaviours of coro1a-Kaede^{+} cells upon apyrase treatment. Time-lapse imaging reveals the dynamic behaviours of coro1a-Kaede^{+} microglial cells in the midbrain after application of control buffer or apyrase at 60 hpf. Time-lapse images are captured for 195 min (1 frame/5 min) and single frame is generated as projections of z stacks of 16 planes with 5 μm distance, covering approximately 80 μm.

Supplementary Movie 4. The neuronal calcium signals of *bax^{cq55}/Tg(HuC:GCaMP6s)* and its sibling. A continuous imaging of one unilateral optic tectum region of 3 dpf *bax^{cq55}/Tg(HuC:GCaMP6s)* and its sibling after injection of Annexin V-Cy5 (magenta). Images are captured at one plane, approximately 30 μm in depth. The imaging duration is 16 min 35 sec (1 frame/5 sec).

Supplementary Movie 5. The neuronal calcium signals in *bax^{cq55}/Tg(HuC:GCaMP6s)* after IP3- and glutamate-uncaging. A continuous imaging of one unilateral optic tectum region of 3 dpf *bax^{cq55}/Tg(HuC:GCaMP6s)* after IP3- and glutamate-uncaging. Images are captured at one plane, approximately 30 μm in depth. The duration of imaging is 16 min 35 sec (1 frame/5 sec).

Supplementary Movie 6. The neuronal calcium signals of *Tg(HuC:GCaMP6s)* after treatment with nemadipine or nilvadipine. A continuous imaging of one unilateral optic tectum region of 3 dpf *Tg(HuC:GCaMP6s)* after the application of nemadipine and nilvadipine. Images are captured at one plane, approximately 30 μm in depth. The duration of imaging is 16 min 35 sec (1 frame/5 sec).

Supplementary Movie 7. The movement behaviours of c-D^{+} cells after treatment with nemadipine. The motility of nemadipine treated c-D^{+} cells is more random and active than those in control group (DMSO). The imaging is started at 3 dpf and lasts 525 min. Images of each group are taken every 5 min. Single frame is generated as projections of z stacks of 16 planes with 5 μm distance, covering approximately 80 μm.
