# Supplementary material for: Brain milieu induces early microglial maturation through the BAX-Notch axis
Source: Nat Commun. 2022 Oct 17;13:6117. doi: 10.1038/s41467-022-33836-2 (PMC9576735; doi:10.1038/s41467-022-33836-2)
Supplement: Supplementary file 11 — Reporting Summary [file 41467_2022_33836_MOESM11_ESM.pdf]

nature portfolio

Corresponding author(s): Li Li; Lingfei Luo

Last updated by author(s): Sep 26, 2022

Reporting Summary

Nature Portfolio wishes to improve the reproducibility of the work that we publish. This form provides structure for consistency and transparency in reporting. For further information on Nature Portfolio policies, see our Editorial Policies and the Editorial Policy Checklist.

Statistics

For all statistical analyses, confirm that the following items are present in the figure legend, table legend, main text, or Methods section.

n/a	Confirmed	
☐	☒	The exact sample size (*n*) for each experimental group/condition, given as a discrete number and unit of measurement
☐	☒	A statement on whether measurements were taken from distinct samples or whether the same sample was measured repeatedly
☐	☒	The statistical test(s) used AND whether they are one- or two-sided<br>*Only common tests should be described solely by name; describe more complex techniques in the Methods section.*
☒	☐	A description of all covariates tested
☒	☐	A description of any assumptions or corrections, such as tests of normality and adjustment for multiple comparisons
☐	☒	A full description of the statistical parameters including central tendency (e.g. means) or other basic estimates (e.g. regression coefficient) AND variation (e.g. standard deviation) or associated estimates of uncertainty (e.g. confidence intervals)
☐	☒	For null hypothesis testing, the test statistic (e.g. *F*, *t*, *r*) with confidence intervals, effect sizes, degrees of freedom and *P* value noted<br>*Give P values as exact values whenever suitable.*
☒	☐	For Bayesian analysis, information on the choice of priors and Markov chain Monte Carlo settings
☒	☐	For hierarchical and complex designs, identification of the appropriate level for tests and full reporting of outcomes
☒	☐	Estimates of effect sizes (e.g. Cohen's *d*, Pearson's *r*), indicating how they were calculated

Our web collection on statistics for biologists contains articles on many of the points above.

Software and code

Policy information about availability of computer code

Data collection	Zeiss Imaging Software (2.6) and Zeiss LSM700 and LSM880 was used for confocal image acquisition.<br>Beckman Coulter Moflo XDP cell sorter was used for flow cytometry.<br>Roche LightCycler 96 Real-Time PCR system was used for Real-Time PCR analysis.<br>LI-COR Odyssey ® XF Imaging System was used for western blotting.
Data analysis	GraphPad Prism (v7.0e), Zeiss Imaging Software (2.6), FlowJo (v10.5.3), LightCycler 96 SW 1.1 Real-Time PCR Analysis, R (4.0), RStudio (1.4.1717), STAR (v2.7.6a), Reference Component Analysis (RCA) (2.0), pheatmap (version 1.0.12), DESeq2 (version 1.32.0), ggplot2 (version 3.3.5), 10xGenomics Cell Ranger (version 5.0.0), Seurat V3, org.Dr.eg.db (3.11.4), DAVID Bioinformatics Resources 6.8 (https://david.ncifcrf.gov/home.jsp), IMARIS (v9.3), Image J (v1.53e), BioRender (https://biorender.com), photoshop CS6, Adobe Illustrator CC 2019.

For manuscripts utilizing custom algorithms or software that are central to the research but not yet described in published literature, software must be made available to editors and reviewers. We strongly encourage code deposition in a community repository (e.g. GitHub). See the Nature Portfolio guidelines for submitting code & software for further information.

Data

Policy information about availability of data

All manuscripts must include a data availability statement. This statement should provide the following information, where applicable:

- Accession codes, unique identifiers, or web links for publicly available datasets
- A description of any restrictions on data availability
- For clinical datasets or third party data, please ensure that the statement adheres to our policy

The data that support the findings of this study, including experimental animals, antibodies, vector constructions, and chemicals, are available within the article, its

Supplementary information, or from the corresponding author upon reasonable request. Data from the bulk and single-cell RNA-seq analyses have been deposited in Sequence Read Archive (SRA) with the accession number PRJNA660936 (https://www.ncbi.nlm.nih.gov/Traces/study/?acc=PRJNA660936) and PRJNA661142 (https://www.ncbi.nlm.nih.gov/Traces/study/?acc=PRJNA661142) and NCBI Gene Expression Omnibus (GEO) with the accession number GSE183340 (https://www.ncbi.nlm.nih.gov/geo/query/acc.cgi?acc=GSE183340) .

Field-specific reporting

Please select the one below that is the best fit for your research. If you are not sure, read the appropriate sections before making your selection.

☒ Life sciences ☐ Behavioural & social sciences ☐ Ecological, evolutionary & environmental sciences

For a reference copy of the document with all sections, see nature.com/documents/nr-reporting-summary-flat.pdf

Life sciences study design

All studies must disclose on these points even when the disclosure is negative.

Sample size	It is generally considered that a sample size containing at least 3 independent replicates can provide adequate statistical power in biochemical analysis. We have described the exact sample size for each experiment in our manuscript.
Data exclusions	No data captured was excluded from the subsequent analyses.
Replication	The exact number of replication for all experiments was described in figure legends and our attempts at replication were successful.
Randomization	Samples and animals were allocated randomly.
Blinding	The experimenters were blinded to the animal genotype, grouping information and data analysis.

Reporting for specific materials, systems and methods

We require information from authors about some types of materials, experimental systems and methods used in many studies. Here, indicate whether each material, system or method listed is relevant to your study. If you are not sure if a list item applies to your research, read the appropriate section before selecting a response.

Materials & experimental systems

n/a	Involved in the study	
☐	☒	Antibodies
☒	☐	Eukaryotic cell lines
☒	☐	Palaeontology and archaeology
☐	☒	Animals and other organisms
☒	☐	Human research participants
☒	☐	Clinical data
☒	☐	Dual use research of concern

Methods

n/a	Involved in the study	
☒	☐	ChIP-seq
☐	☒	Flow cytometry
☒	☐	MRI-based neuroimaging

Antibodies

Antibodies used	The following antibodies were used for immunoblotting or immunostaining: goat anti-GFP (Abcam, #ab6658, 1:400), mouse anti-DsRed (Santa Cruz, #sc-101526,1:400), rabbit anti-DsRed (Takara Bio Clontech, 632496, 1:400), rabbit anti-Lcp1 (GTX124420, GeneTex, 1:400), mouse anti-zBax (This paper, 1:400), mouse anti-Bax (B-9) (Santa Cruz, #sc-7480, 1:200), rabbit anti-SOX2 (Abcam, #ab94959, 1:200), rabbit anti-CaMKII (pan) (Cell Signalling technology, #4436, 1:400), rabbit anti-phospho-CaMKII (Thr286) (Cell Signalling technology, #12716, 1:400), rabbit anti-CREB (Abcam, #ab32515,1:400), rabbit anti-CREB (phospho S133) (Abcam, #ab32096,1:400), rabbit anti-NICD1a (Abcam, #ab83232, 1:400), rabbit anti-DLL3 (Abcam, #ab103102, 1:200), rabbit β-catenin (Merck Millipore, #06-734, 1:400), Rat anti-F4/80 (Abcam, #ab6640, 1:100), goat anti-IBA1 (Abcam, #ab5076, 1:200), mouse anti-Hes1 (Abcam, #ab119776, 1:400), rabbit anti-activated Notch1 (Abcam, #ab8925, 1:400), rabbit anti-Rbpj (BOSTER, #A00767-1, 1:400), rabbit Anti- Active Caspase-3 (BD Biosciences, #559565,1:400), mouse anti-PCNA (Sigma-Aldrich, P8825, 1:800), mouse anti-FLAG (Sigma-Aldrich, #F1804, 1:400), mouse anti-β tubulin (Thermo Fisher Scientific, #MA5-11732, 1:1000), FITC anti-mouse/human CD11b antibody (Elabscience, #E-AB-F1081C, 1:20), Alexa Fluor 700 anti-mouse CD45 antibody (BioLegend, #103128, 1:50), 488 Goat Anti-Rat IgG (Abcam, ab150165, 1:400), Alexa Fluor 488 Donkey Anti-Goat IgG (Thermo Fisher Scientific, #A-11055, 1:400), Alexa Fluor 555 Donkey Anti-Rabbit IgG (Thermo Fisher Scientific, #A-31572, 1:400), Alexa Fluor 555 Donkey Anti-Mouse IgG (Thermo Fisher Scientific, #A-31570, 1:400), Alexa Fluor 647 Donkey Anti-Mouse IgG (Thermo Fisher Scientific, #A-31571, 1:400), Goat anti-Mouse IgG (Thermo Fisher Scientific, #31430, 1:1000), and Goat anti-Rabbit IgG (Thermo Fisher Scientific, #31460, 1:1000)
Validation	All antibodies except for mouse anti-zBax were commercial in origin and validated by the company. Information regarding validation processes can be easily accessed on the company's web site using the product numbers listed above. Additionally, we validated antibodies: rabbit anti-NICD1a (Abcam, #ab83232, 1:400), rabbit anti-DLL3 (Abcam, #ab103102, 1:200), rabbit anti-CREB (phospho S133) (Abcam, #ab32096, 1:400), and rabbit anti-phospho-CaMKII (Thr286) (Cell Signaling Technology, #12716, 1:400), by western

	blot. The validation results were presented in Supplementary fig. 2d, 4e, 5i, 7a, and 7h. The related description was included in the Methods section. The detailed information about a monoclonal antibody, mouse anti-zBax, against zebrafish Bax antigen has been provided in our Methods section. The appropriate dilution for all the antibodies was determined through preliminary experiments.

Animals and other organisms

Policy information about studies involving animals; ARRIVE guidelines recommended for reporting animal research

Laboratory animals	Zebrafish embryos were kept at a constant temperature (28.5°C) on an incubator.<br>According to the purpose of each experiment, different transgenic and mutant zebrafish lines were used in this study: Tg(coro1a:DsRed), Tg(coro1a:Kaede), Tg(mfap4:GFP), Tg(apoeb:GFP), Tg(Tp1bglob:eGFP), Tg(Tp1bglob:hmgb1-mCherry), Tg (hsp70:DN-MAML-GFP), Tg(HuC:GCaMP6s), Tg(HuC:GFP), Tg(HuC:dlb), Tg(NBT:DenNTR), Tg(NBT:creb1a), Tg(NBT:bax), Tg (hsp70:bax), Tg(coro1a:bax), Tg(coro1a:NICD1a), Tg(ubiq:secAnnexinV-mVenus), and Tg(coro1a:DN-MAML-FLAG), mibta52b mutant, baxacq55 mutant, baxΔ1/Δ1 mutant, baxΔ58/Δ58 mutant, and dlb-/- mutant. AB and WIK strains were used as control and as a background line for experiments. 52 hpf, 55 hpf, 2 dpf, 2.5 dpf, 3 dpf, 4 dpf, and 6 dpf zebrafish were used for experiments.<br><br>Mice were bred at 23°C, with 40-60% relative humidity, 12hrs light cycle (8am-8pm).<br>According to the purpose of each experiment, WT C57BL/6J (The Jackson Laboratory, #000664), Bax+/tm1Sjk (The Jackson Laboratory, #002994), RBP-J(f/f) (The Jackson Laboratory Stock, #034200) and Cx3cr1-Cre (The Jackson Laboratory Stock, #025524) mice were used and both genders were used in the entire study. E12.5 and E14.5 mice embryos was used.
Wild animals	No wild animals involved in this study.
Field-collected samples	The study did not involve samples collected from the field.
Ethics oversight	All animal experiments were approved by the Institutional Review Board of Southwest University (Chongqing, China).

Note that full information on the approval of the study protocol must also be provided in the manuscript.

Flow Cytometry

Plots

Confirm that:

- [x] The axis labels state the marker and fluorochrome used (e.g. CD4-FITC).
- [x] The axis scales are clearly visible. Include numbers along axes only for bottom left plot of group (a 'group' is an analysis of identical markers).
- [x] All plots are contour plots with outliers or pseudocolor plots.
- [x] A numerical value for number of cells or percentage (with statistics) is provided.

Methodology

Sample preparation	Zebrafish microglia isolation: The green-kaede+ cells in 3 dpf siblings and Bax cq55 Tg(coro1a:Kaede) zebrafish brain were labeled, and whole brain were dissected for isolating labeled coro1a-kaede+ cells to do bulk RNA seq and RNA extraction by REPLI-g WTA Single Cell Kit (QIAGEN, #150063). The brain of 4 dpf WT Tg(coro1a:DsRed) zebrafish was dissected for DsRed+ microglia isolation to do single cell RNA seq.<br>Zebrafish neuron isolation: The whole brain of 3 dpf siblings and Bax cq55 Tg(HuC:GFP) zebrafish brain were dissected to isolate GFP+ neurons to do bulk RNA seq and RNA extraction by REPLI-g WTA Single Cell Kit (QIAGEN, #150063). The whole brain of 55 hpf WT Tg(HuC:GFP) zebrafish brain were dissected to isolate GFP+ neurons to perform transplantation assay.<br>To prepare single cell suspensions. Zebrafish were anesthetized with 0.02% tricaine. 200 brains were freshly isolated in ice-cold dulbecco's phosphate-buffered saline (dPBS) and incubated with papain (10U/mL) at 30°C for 20 min. After centrifuging with 800 rpm and a brief washing, the digested tissues were re-suspended with 5% FBS (dissolved in dPBS) and passed through 1.2 µm and 0.5 µm glass pipette 7 times, respectively. Then, the samples were filtered through a 40 µm Cell Strainer to gain single cell suspension for FACS sorting.<br>Mice microglia isolation: Whole E12.5 or E14.5 mouse embryonic brains, excluding olfactory bulbs, cerebella and spine cords, were dissected for microglia isolation. The brains were minced to ~1mm3 with scissors under a dissecting microscope. Brain tissues were digested by papain at 37°C for around 40 min, and then centrifuged with 800 rpm at 4°C for 5 min. Cell pellets were washed by dBSS, resuspended in 5 mL 5% FBS, passed through 1.2 µm and 0.5 µm glass and pushed through a 40 µm cell strainer. Cell suspensions were stained with CD11b antibody for FACS sort to do primary microglia culture in vitro. Cell suspensions were stained with CD11b and CD45 antibody for FACS analysis and RNA extraction by REPLI-g WTA Single Cell Kit (QIAGEN, #150063).
Instrument	Beckman Coulter Moflo XDP cell sorter
Software	FlowJo Software (10.5.3) of Beckman Coulter Moflo XDP cell sorter was used for cell sorting.
Cell population abundance	About 3x10^4 coro1a-DsRed+ cells in zebrafish larval brains were collected by Moflo XDP cell sorter with 99% of purity and prepared to conduct single-cell sequencing. About 500 HuC-GFP+ neurons and 100 photo-converted red coro1a-Kaede+ microglial cells in zebrafish larval brains were sorted by Beckman Coulter Moflo XDP cell sorter with 99% of purity, to isolate total RNA by REPLI-g WTA Single Cell Kit and establish the cDNA libraries by Anoroda corporation for Bulk RNA Sequencing. About 1x10^6 HuC-GFP+ cells in zebrafish larval brains were collected by Beckman Coulter Moflo XDP cell sorter with 99% of

purity and prepared to perform zebrafish transplantation. About 6x10^4 CD11b+ primary microglia were isolated from E12.5 embryonic mice cerebral cortexes (largely immature microglia at embryonic stages) by Beckman Coulter Moflo XDP cell sorter with 95% of purity and prepared for culture in vitro. About 4x10^4 CD11bhiCD45lo E14.5 mouse brains microglia were sorted by Beckman Coulter Moflo XDP cell sorter with 99% of purity and prepared for FACS analysis. About 1000 CD11bhiCD45lo E14.5 mouse brains microglia were sorted by Beckman Coulter Moflo XDP cell sorter with 99% of purity and prepared for RNA extraction by REPLI-g WTA Single Cell Kit.

Gating strategy

Gating strategy for microglia isolated from 4 dpf WT Tg(coro1a:DsRed) transgenic zebrafish brain samples as presented in Supplementary Fig. 4f. Samples of mouse brains microglia were gated accordingly to specific gene markers (CD11bhiCD45lo), refer to Methods for details. Gating strategies for FACS sorted cells are shown in Supplementary fig. 8e and 10a.

☒ Tick this box to confirm that a figure exemplifying the gating strategy is provided in the Supplementary Information.
