# Supplementary material for: Author Correction: Brain milieu induces early microglial maturation through the BAX-Notch axis
Source: Nat Commun. 2025 Apr 7;16:3296. doi: 10.1038/s41467-025-58603-x (PMC11977181; doi:10.1038/s41467-025-58603-x)
Supplement: Supplementary file 1 — Updated Supplementary Information [file 41467_2025_58603_MOESM1_ESM.pdf]

Supplementary information

Brain milieu induces early microglial maturation through the BAX-Notch axis

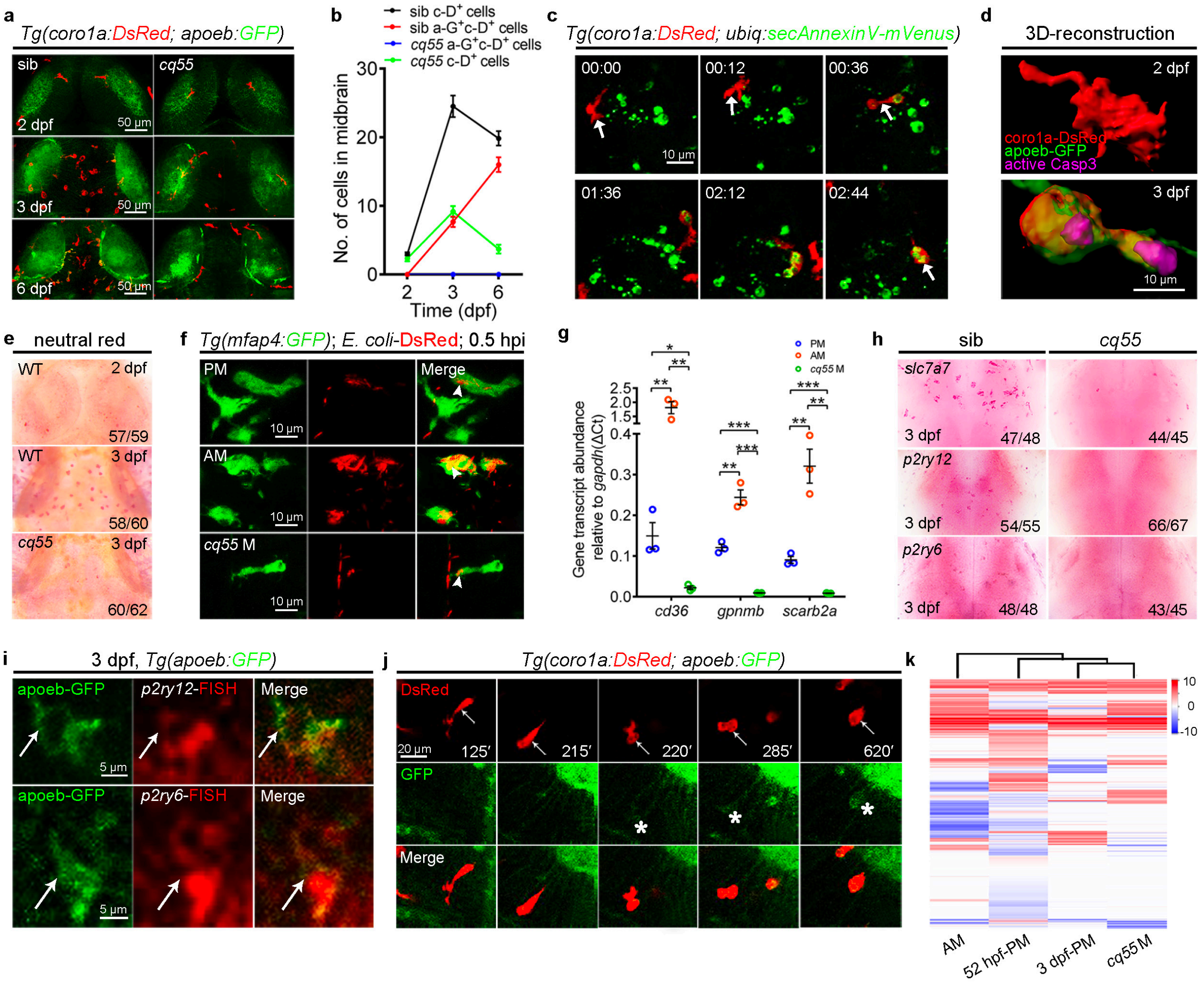

Supplementary Figure 1. The zebrafish *cq55* mutants exhibit defective microglial development. a Fluorescent images of microglial cells in the midbrain of *Tg(coro1a:DsRed;apoeb:GFP)* zebrafish at 2 dpf, 3 dpf, and 6 dpf. **b** Quantification of c-D$^+$ and a-G$^+$c-D$^+$ cells in **a** (2 dpf: sib: $n = 9$, *cq55*: $n = 6$; 3 dpf: sib: $n = 6$, *cq55*: $n = 6$; 6 dpf: sib: $n = 7$, *cq55*: $n = 10$). **c** Confocal time-lapse imaging of one c-D$^+$ cell (white arrow) gradually engulfing the ubiq-secAnnexinV-mVenus$^+$ signals and showing an amoeboid morphology at 52 hpf. **d** 3D reconstructions of coro1a-DsRed (red), apoeb-GFP (green), and active Casp3 (magenta) signals in 2 dpf PM (top) and 3 dpf AM (bottom) from confocal z-stacks. **e** Neutral red staining of the midbrains. **f** Fluorescent images of the DsRed$^+$ *E. coli* phagocytosed by mfap4-GFP$^+$ PM, AM, and *cq55* M at 0.5 hpi. White arrowheads indicate red bacterial inclusions are inside the mfap4-GFP$^+$ cells. **g** Transcriptional levels of phagocytosis related genes, including *cd36*, *gpnmb*, and *scarb2a* in PM, AM, and *cq55* M. The data are from three independent experiments. Each

dot represents an independent experiment. **h** WISH of *slc7a7*, *p2ry12*, and *p2ry6* in 3 dpf midbrains of siblings and *cq55* mutants. **i** Fluorescent images of FISH and anti-GFP staining of apoeb-GFP (green), *p2ry12* (red), or *p2ry6* (red) in 3 dpf midbrains. White arrowheads indicate the co-expression of apoeb-GFP and *p2ry12* or *p2ry6*. **j** Confocal time-lapse imaging of one coro1a-DsRed$^+$ cell (white arrow) gradually expressing the apoeb-GFP (white asterisk) and showing an amoeboid morphology at 52 hpf. **k** Heat-map results of all differentially expressing genes in 52 hpf PM, 3 dpf PM, 3 dpf AM, and 3 dpf *cq55* M. c-D, coro1a-DsRed; a-G, apoeb-GFP; dpf, days post-fertilization; hpi, hours post-injection; hpf, hours post-fertilization; sib, siblings of *cq55* mutants; FISH, fluorescence *in situ* hybridization. Numbers in the right corners in **e** and **h** indicate the counts of embryos with a typical appearance (first number) in the total pools (last number). Error bars, mean ± SEM. *$P < 0.05$; **$P < 0.01$; ***$P < 0.001$; Unpaired two-tailed Student's t test. Source data are provided as a Source Data file.

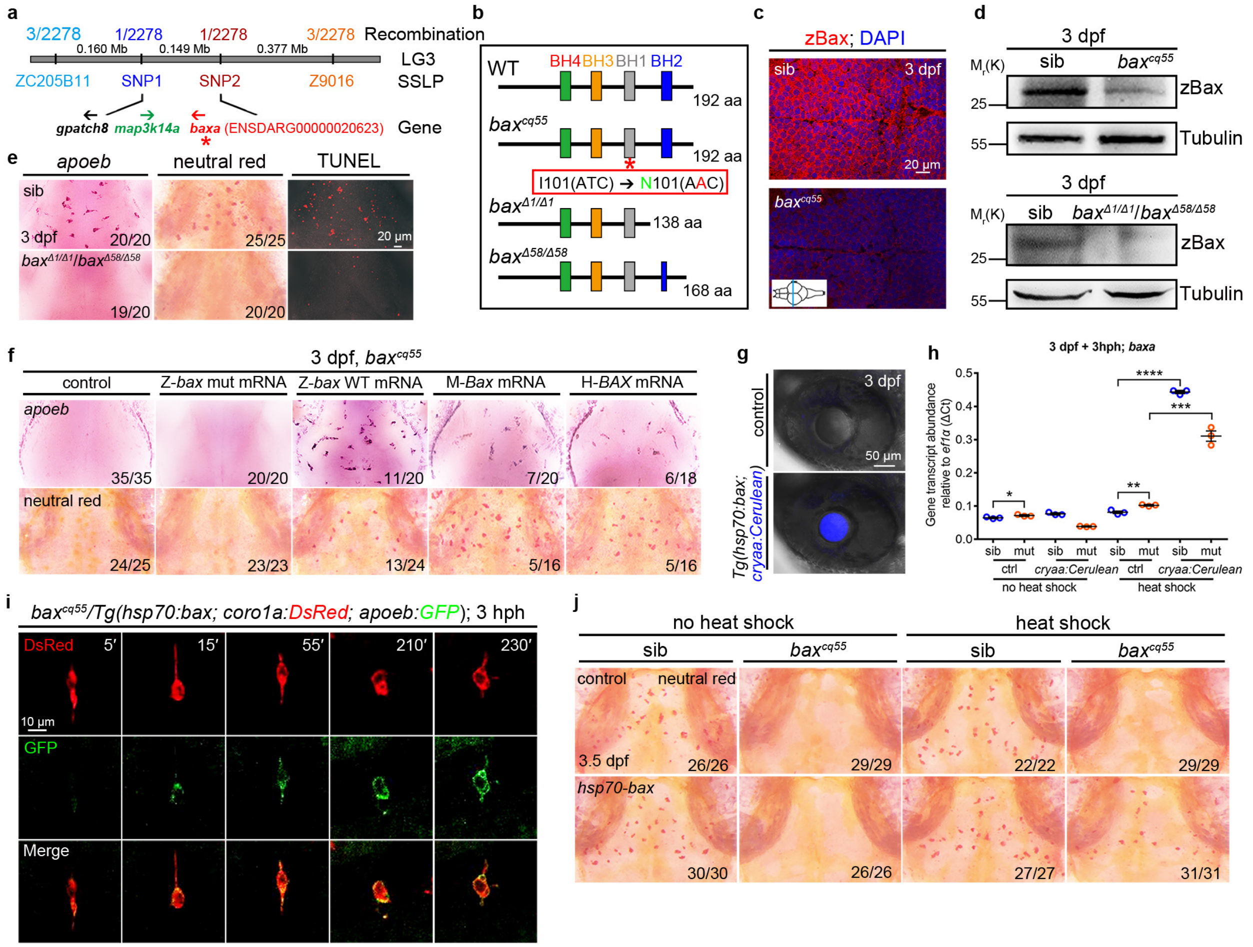

Supplementary Figure 2. *bax* is cloned in *cq55* mutants. a Genetic mapping of the candidate mutation (*baxa*, red asterisk) in the *cq55* mutants. The mutation was positioned on LG3. Numbers of recombinants were depicted in the top lane. SSLP and SNP markers used for mapping are in the middle lane. The genes in the targeted regions are annotated in the bottom lane. **b** The presumed protein fragments in different mutant alleles. T to A mutation in bax^{cq55} mutants caused the alteration of Ile (I) to Asn (N) in the conserved BH1 domain of Bax protein. The newly generated $bax^{\Delta 1/\Delta 1}$ and $bax^{\Delta 58/\Delta 58}$ mutants were presumed to format truncated Bax proteins. **c** Immunofluorescence of zBax in siblings and bax^{cq55} transverse sections of 3 dpf midbrains. **d** WB of zBax in the total proteins from the bax^{cq55}, $bax^{\Delta 1/\Delta 1}$ and $bax^{\Delta 58/\Delta 58}$ brains. The experiment was repeated three times and the results were similar. **e** Representative images of *apoeb* WISH, NR and TUNEL staining in the midbrains of 3 dpf siblings, $bax^{\Delta 1/\Delta 1}$ and $bax^{\Delta 58/\Delta 58}$ mutants. **f** Representative images of *apoeb* WISH and NR staining in 3 dpf midbrains of bax^{cq55} mutants injected with Z-*bax* mut mRNA, Z-*bax* WT mRNA, M-*Bax* mRNA, and H-*BAX* mRNA. **g** Fluorescent images of the *cryaa*-Cerulean (blue) in 3 dpf *Tg(hsp70:bax;cryaa:Cerulean)* zebrafish embryos retina. **h** qPCR result of *bax* transcripts in 3 dpf *Tg(hsp70:bax;cryaa:Cerulean)* at 3 hph. The data were from three independent experiments. Each dot represents an independent experiment. **i** Confocal time-lapse imaging of one

coro1a-DsRed$^+$ cell gradually expressing the apoeb-GFP and showing an amoeboid morphology in the heat-shocked *bax^{cq55}/Tg(hsp70:bax;cryaa:Cerulean)* embryos. **j** Representative images of NR staining in 3.5 dpf sib and *bax^{cq55}/Tg(hsp70:bax;cryaa:Cerulean)* transgene midbrains. LG3, linkage group 3; SSLP, simple sequence length polymorphism; SNP, single nucleotide polymorphism; WB, Western blotting; Z, zebrafish; M, mouse; H, human; hph, hours post heat shock. Numbers in the right corners in **e**, **f**, and **j** indicate the counts of embryos with a typical appearance (first number) in the total pools (last number). Error bars, mean ± SEM. *$P < 0.05$; **$P < 0.01$; ***$P < 0.001$; ****$P < 0.0001$; Unpaired two-tailed Student's t test. Source data are provided as a Source Data file.

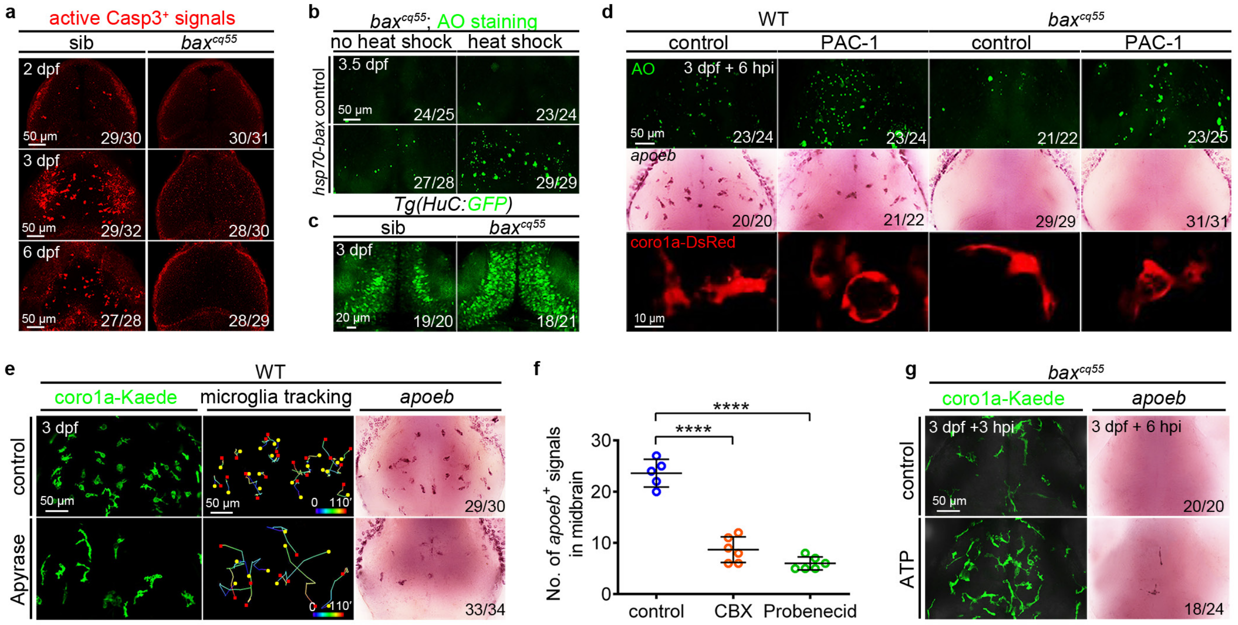

Supplementary Figure 3. Nucleosides act as the mediators of apoptotic neurons to position microglia. a Immunofluorescence of active Casp3 (red) in 2 dpf, 3 dpf and 6 dpf midbrains of siblings and *bax*^{cq55} mutants. **b** Fluorescent images of AO staining in the midbrains of *bax*^{cq55} (top) and/or *bax*^{cq55}*/Tg(hsp70:bax)* (bottom) with or without heat-shock. **c** Fluorescent images of H-G^{+} neurons in 3 dpf midbrains of siblings and *bax*^{cq55} mutants. **d** Fluorescent images of AO staining (top), *apoeb* WISH (middle), and c-D^{+} cell morphology (bottom) in the midbrains of WT and/or *bax*^{cq55} mutants treated with control buffer and/or PAC-1. **e** Fluorescent images of c-K^{+} cells (left), the trajectory of c-K^{+} cells (middle) and *apoeb* WISH (right) after 12 hours of treatment with or without apyrase. Each line represents one cell. The start and end points of individual tracks are marked by the yellow circle and red square, respectively. **f** Quantification of *apoeb*$^{+}$ signals in WT midbrains after treatment with control buffer, CBX and Probenecid (control buffer: n = 5, CBX: n = 6, Probenecid: n = 6). Each dot represents one fish. **g** Fluorescent images of c-K^{+} cells and *apoeb* WISH in the midbrains after injection with control buffer and/or ATP. AO, acridine orange; hpi, hours post injection. Numbers in the down-right corners in **a**, **b**, **c**, **d**, **e**, and **g** indicate the counts of embryos with a typical appearance (first number) in the total pools (last number). Error bars, mean ± SEM. ****P < 0.0001; Unpaired two-tailed Student's t test. Source data are provided as a Source Data file.

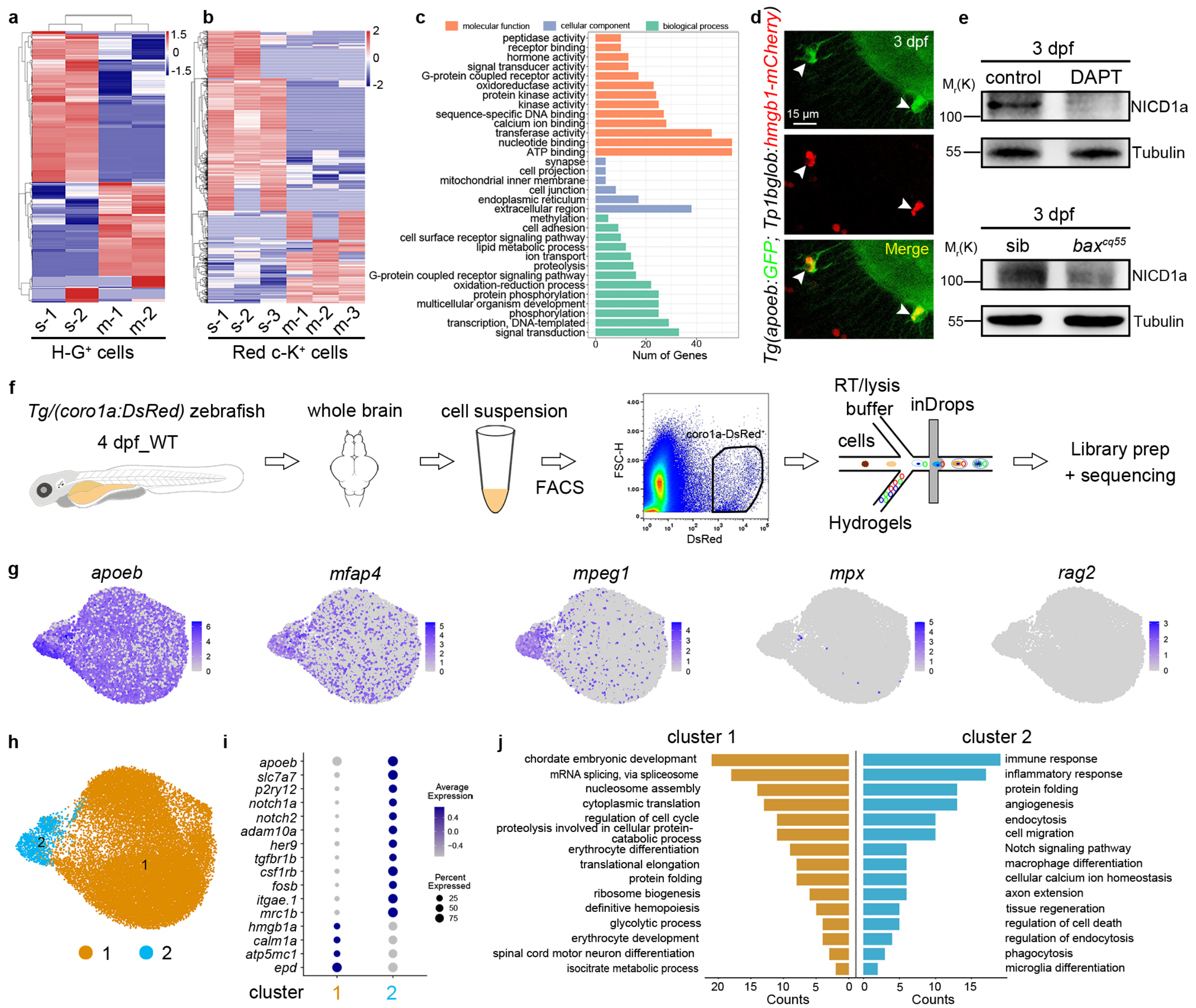

Supplementary Figure 4. Notch signalling enriches in mature microglia. **a**, **b** Heat-map results of all differentially expressing genes in H-G^{+} neurons (**a**) and red c-K^{+} microglial cells (**b**) between 3 dpf siblings and *bax^{cq55}* mutants. **c** GO terms enriched in *bax^{cq55}* versus sibling H-G^{+} neurons. **d** Fluorescent images of apoeb-GFP^{+} microglia (white arrowheads) co-localized with Tp1bglob-hmgb1-mCherry in 3dpf midbrains. **e** WB of NICD1a using the brain proteins. The presumed molecular size of NICD1a is about 80 kDa; Tubulin is a loading control. The experiments were repeated three times and the results were similar. **f** The experimental workflow of scRNA-seq, created with BioRender.com. **g** *t*SNE visualization of the microglia markers (*apoeb*, *mfap4*, *mpeg1*), granulocyte marker (*mpx*), and T lymphocyte marker (*rag2*). The colour key indicates the expression levels. **h** Visualization of microglia clusters via *t*-SNE. **i** The features of typical genes in two microglia clusters in **h**. **j** GO terms of the genes enrichment in each microglia subtype. The counts indicated the gene numbers in a GO pathway that were differentially expressed in each subtype. s, sibling; m, mutant; scRNA-seq, single-cell RNA-sequencing; *t*-SNE, *t*-distributed stochastic neighbor embedding; GO, Gene ontology. Source data are provided as a Source Data file.

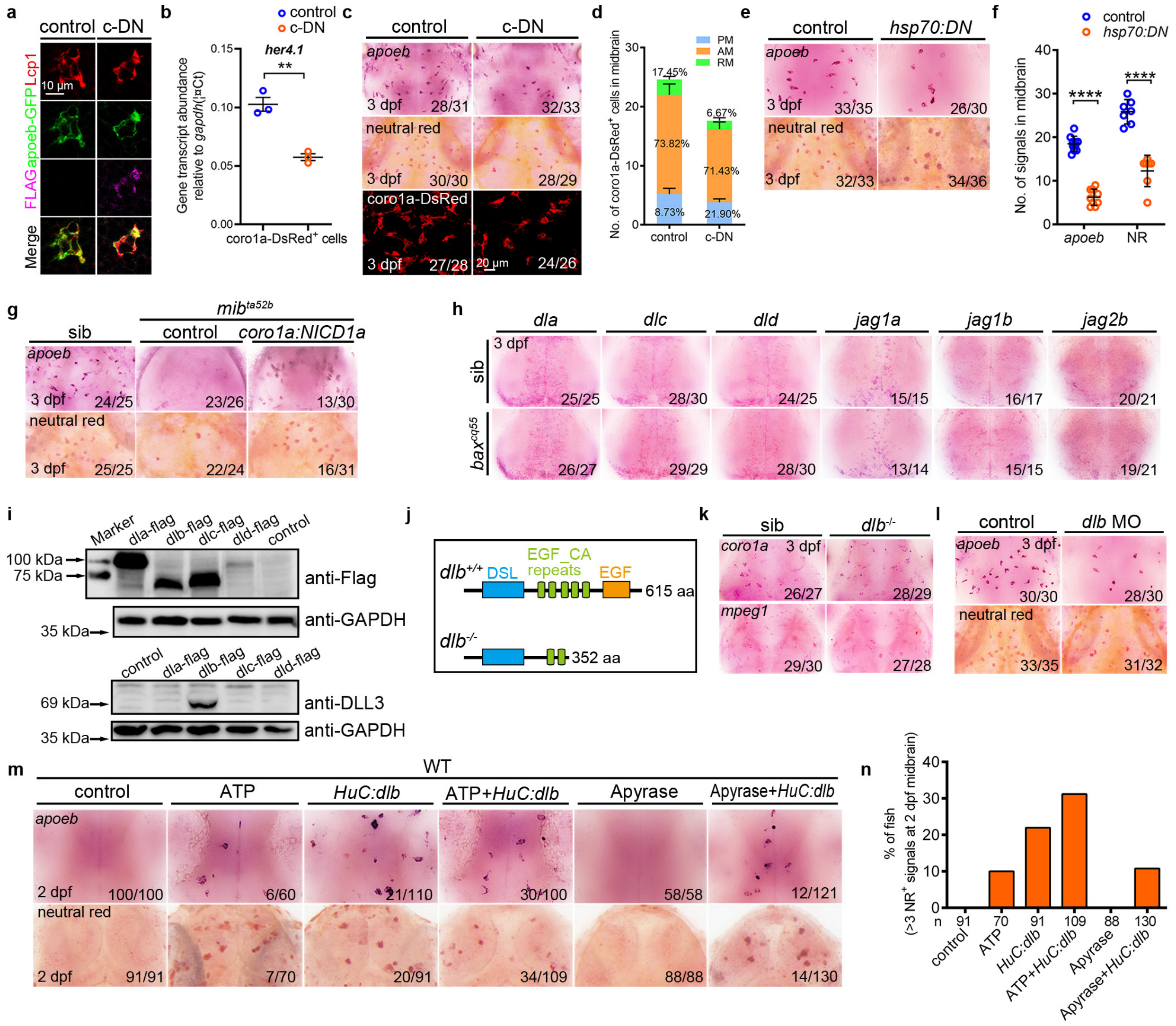

Supplementary Figure 5. Notch signalling regulates microglial maturation. a Immunofluorescence of Lcp1 (red), GFP (green), and FLAG (magenta) in 3 dpf control and c-DN transgenic midbrains. **b** qPCR result of *her4.1* transcriptional levels in the c-D^{+} cells sorted from 3 dpf control and c-DN transgenic brains. The data are from three independent experiments. Each dot represents an independent experiment. **c** Representative images of *apoeb* WISH, NR staining and c-D^{+} signals in 3dpf control and c-DN transgenic midbrains. **d** The numbers and percentages of PM, AM, and RM in 3dpf control and c-DN transgenic midbrains (n = 5). **e**, **f** Representative images (**e**) and quantification (**f**, *apoeb*: control: n = 10, *hsp70:DN*: n = 9; NR: n = 7) of *apoeb* WISH and NR staining in 3 dpf control and *hsp70:DN* midbrains. Each dot represents one fish. **g** Representative images of *apoeb* WISH and NR staining in *mib*^{ta52b} mutants after supplying *coro1a:NICD1a*. **h** Representative images of *dla*, *dlc*, *dld*, *jag1a*, *jag1b*, and *jag2b* WISH in 3 dpf siblings and *bax*^{cq55} mutants. **i** WB of FLAG and DLL3 in the HEK293T cell lysates transiently transfected with pCS2(+), pCS2(+)-*dla-flag*, pCS2(+)-*dlb-flag*, pCS2(+)-*dlc-flag*, and pCS2(+)-*dld-flag* plasmids. GAPDH is shown as a loading control. The

presumed molecular sizes of targeted proteins: pCS2(+)-*dla-flag*, 87 kDa; pCS2(+)-*dlb-flag*, 70 kDa; pCS2(+)-*dlc-flag*, 74 kDa; pCS2(+)-*dld-flag,* 81 kDa. The experiment was repeated three times. **j** The presumed protein fragments of $dlb^{-/-}$ mutants. **k** Representative images of *apoeb* WISH and NR staining in 3 dpf siblings and $dlb^{-/-}$ mutant midbrains. **l** Representative images of *apoeb* WISH and NR staining in 3 dpf control and *dlb* MO midbrains. **m** Representative images of *apoeb* WISH and NR staining after injection with control buffer, ATP, *HuC:dlb*, ATP plus *HuC:dlb*, Apyrase, Apyrase plus *HuC:dlb*. **n** The percentage of zebrafish embryos with NR^+ signals > 3 in **m**. c-DN, coro1a-DN-MAML-FLAG; *hsp70:DN*, *Tg(hsp70:DN-MAML-GFP)*; MO, morpholino. Numbers in the down-right corners in **c**, **e**, **g**, **h**, **k**, **l**, and **m** indicate the counts of embryos with a typical appearance (first number) in the total pools (last number). Error bars, mean ± SEM. $^{**}P < 0.01$; $^{****}P < 0.0001$; Unpaired two-tailed Student's t test. Source data are provided as a Source Data file.

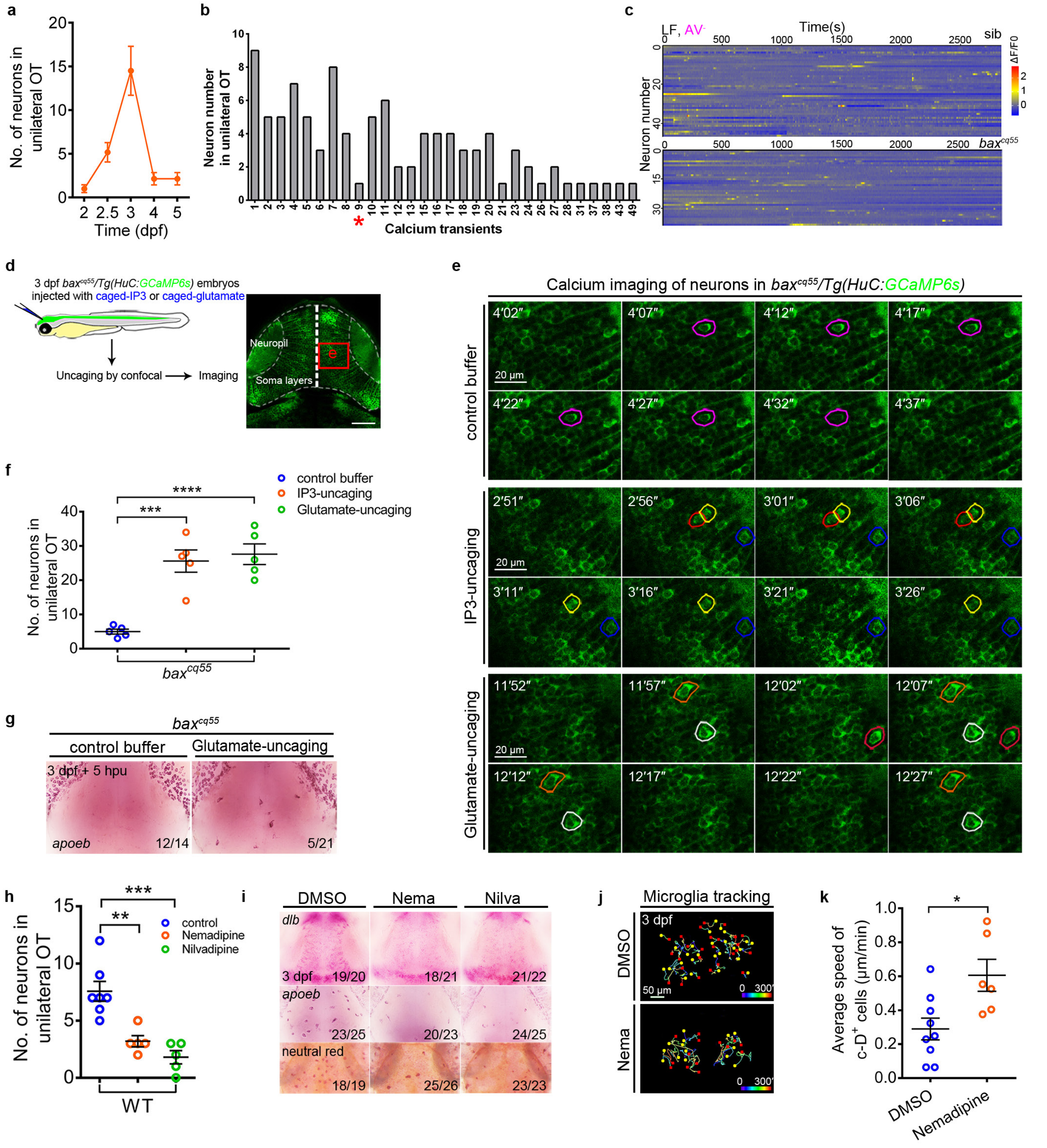

Supplementary Figure 6. BAX regulates microglia signatures through neuronal activities. a Quantification of neurons with calcium events in the unilateral midbrains of *Tg(HuC:GCaMP6s)* embryos at 2 dpf (n = 6), 2.5 dpf (n = 6), 3 dpf (n = 8), 4 dpf (n = 7), and 5 dpf (n = 7). **b** The distribution of varied Ca^{2+} transients in every neuronal Ca^{2+} event (total n = 99 neurons). Red asterisk indicates that 9 times is set as the threshold to separate the low- and high-frequency. **c** Low-frequency Ca^{2+} traces of all AV^{-} active neurons (siblings: n = 46 in 6 embryos; *bax*^{cq55}: n = 38 in 6 embryos). **d** The schematic diagram of IP3- and glutamate-uncaging in

bax^{cq55}*/Tg(HuC:GCaMP6s)* unilateral midbrains, created with photoshop CS6. The red square is the confocal time-lapse imaging site in **e**. **e** Confocal time-lapse imaging of evoked neuronal calcium transients (circles with different colors) after uncaging of control buffer (top), IP3 (middle) or glutamate (bottom) in 3 dpf *bax*^{cq55}*/Tg*(*HuC:GCaMP6s*) larval brains (n = 5). **f** The numbers of active neurons with calcium events in the unilateral OT according to **e** (n = 5). Each dot denotes one fish. **g** Representative images of *apoeb* WISH in 3 dpf *bax*^{cq55} mutants midbrains after glutamate uncaging. **h** The numbers of active neurons with calcium events in the unilateral OT after nemadipine and nilvadipine treatment (control: n = 7, nemadipine and nilvadipine: n = 5). Each dot denotes one fish. **i** Representative images of *dlb* and *apoeb* WISH, and NR staining in 3 dpf DMSO-, nemadipine-, and nilvadipine-treated zebrafish midbrains. **j** Microglial cells movement trajectories during 145 min in *Tg*(*coro1a:DsRed*) larval midbrain treated with DMSO and nemadipine. The starting point of individual tracks is marked by yellow circles and the end point by red squares. **k** The average movement speed (μm/min) of microglial cells in **j** (control: n = 9, nemadipine: n = 6). Each dot represents one c-D^{+} cell. OT, optic tectum; LF, low frequency; AV, Annexin V; Nema, nemadipine; Nilva, nilvadipine. Error bars, mean ± SEM. **P* < 0.05; ***P* < 0.01; ****P* < 0.001; *****P* < 0.0001; Unpaired two-tailed Student's t test. Source data are provided as a Source Data file.

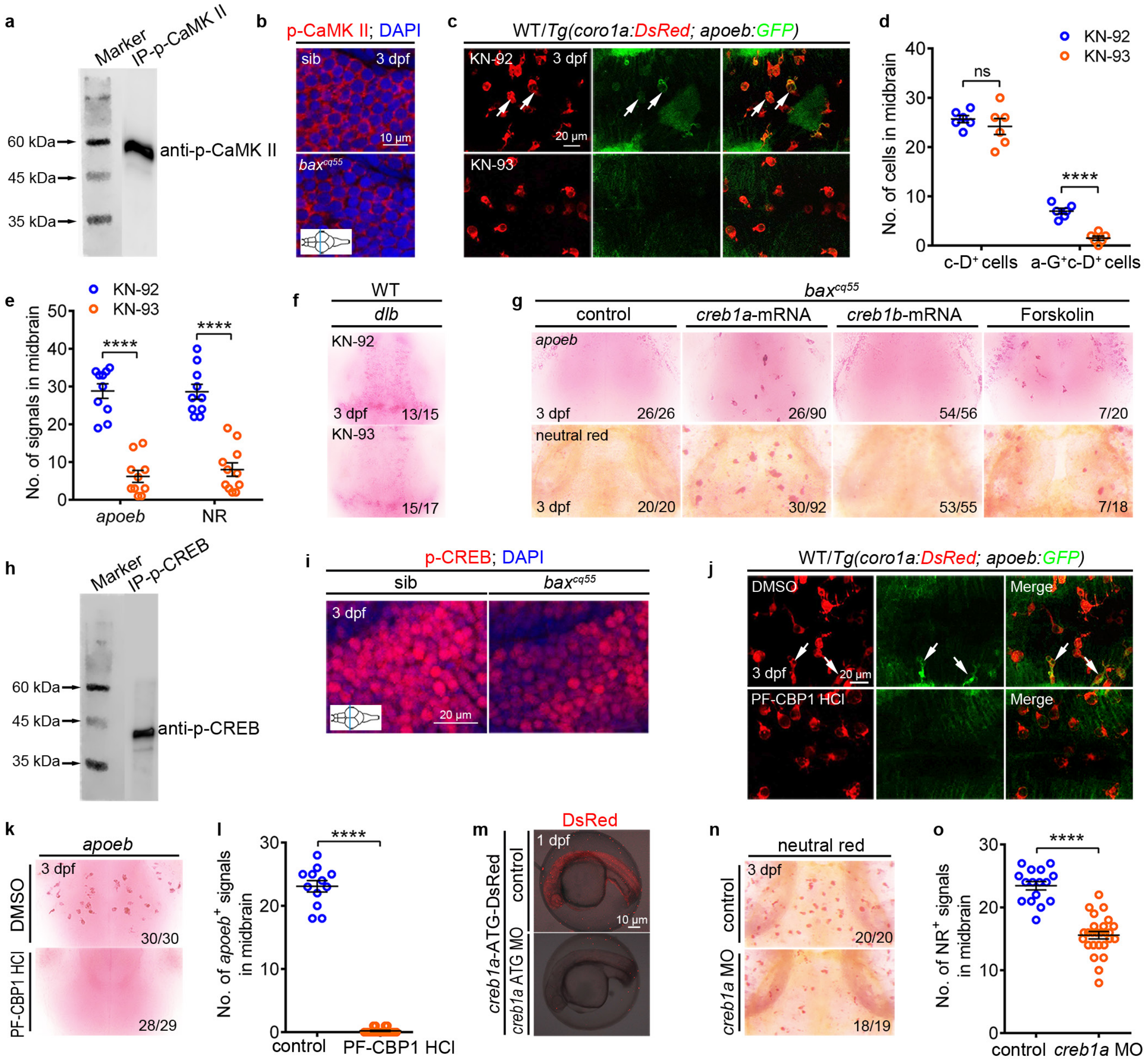

Supplementary Figure 7. The CaMKII-CREB axis is essential for microglial differentiation. **a** WB of p-CaMKII (60 kDa) in 3 dpf brain protein immunoprecipitated by the p-CaMKII antibody. The experiment was repeated three times. **b** Immunofluorescence of p-CaMKII in the transverse sections of 3 dpf siblings and *bax*^{cq55} midbrains. **c** Fluorescent images of c-D^{+} and a-G^{+} cells in 3 dpf *Tg*(*coro1a:DsRed;apoeb:GFP*) midbrains treated with KN-92 or KN-93. White arrows indicate the a-G^{+}c-D^{+} cells. **d**, **e** The number of c-D^{+} and a-G^{+}c-D^{+} cells (**d**, $n = 6$) and *apoeb*$^{+}$ and NR^{+} signals (**e**, *apoeb*: KN-92: $n = 9$, KN-93: $n = 10$; NR: KN-92: $n = 9$, KN-93: $n = 11$) in 3 dpf midbrains treated with KN-92 and KN-93. **f** Representative images of *dlb* WISH in 3 dpf midbrains treated with KN-92 and KN-93. **g** Representative images of *apoeb* WISH and NR staining in 3 dpf *bax*^{cq55} midbrains injected with *creb1a*, *creb1b* mRNA, or treated with Forskolin. **h** WB of p-CREB (37 kDa) in 3dpf brain protein immunoprecipitated by the p-CREB antibody. The experiment was repeated three times. **i** Immunofluorescence of p-CREB in the transverse sections of 3 dpf siblings and *bax*^{cq55} midbrains. **j** Fluorescent images of c-D^{+} and

a-G$^+$ cells in 3 dpf midbrains of *Tg*(*coro1a:DsRed;apoeb:GFP*) embryos treated with control and PF-CBP1 HCL. White arrows indicate the c-D$^+$a-G$^+$ cells. **k**, **l** Representative images (**k**) and quantification (**l**, control: n = 12, PF-CBP1 HCl: n = 20) of *apoeb*$^+$ signals in 3 dpf midbrains treated with control and PF-CBP1 HCl. **m** Fluorescent images of DsRed in 1 dpf control and *creb1a* ATG MO embryos injected with *creb1a*-ATG-DsRed mRNA. **n**, **o** Representative images (**n**) and quantification (**o**, control: n = 16, *creb1a* MO: n = 26) of NR$^+$ signals in 3 dpf control and *creb1a* MO midbrains. Numbers in the down-right corners in **f**, **g**, **k**, and **n** indicate the counts of embryos with a typical appearance (first number) in the total pools (last number). Each dot in **d**, **e**, **l**, and **o** represents one fish. Error bars, mean ± SEM. ****$P < 0.0001$; ns, no significant, Unpaired two-tailed Student's t test. Source data are provided as a Source Data file.

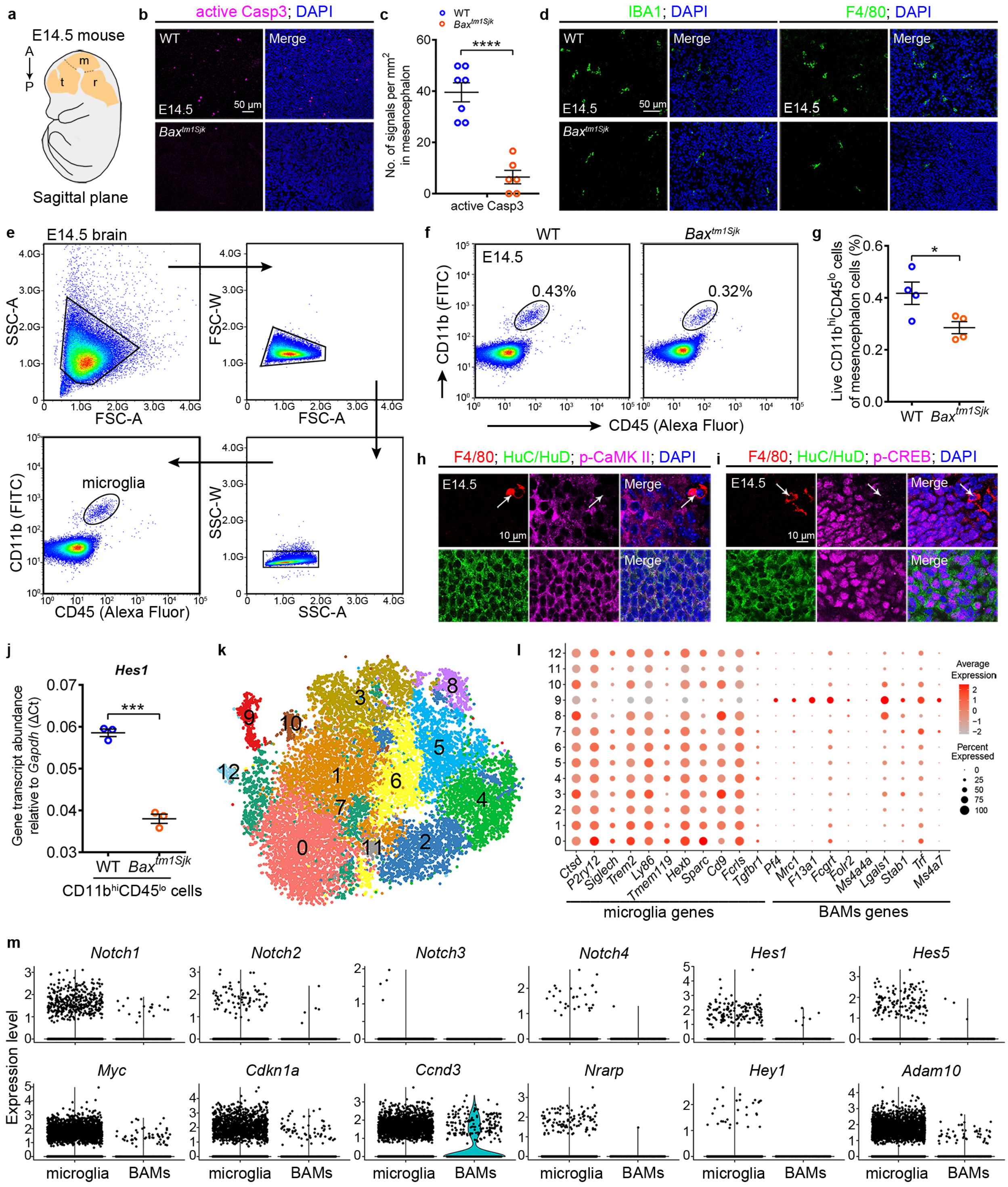

Supplementary Figure 8. The regulatory mechanism of Bax-CaMKII-CREB axis is involved in mice microglial development. a Schematic view of the E14.5 mice brain sagittal section, created with photoshop CS6. Fluorescent images in Figures 6 and 7 are captured from mesencephalon region. t, telencephalon; m, mesencephalon; r, rhombencephalon. **b**, **c** Immunofluorescence (**b**) and quantification (**c**) of active Casp3 (magenta) and DAPI (blue)

signals in the sagittal sections of E14.5 WT and *Bax*^{tm1Sjk} mice mesencephalon (WT, *n* = 7 mice; *Bax*^{tm1Sjk}, *n* = 6 mice). Each symbol represents one mouse. Three sections per mouse were quantified. **d** Immunofluorescence of IBA1^{+} (left) and F4/80^{+} (right) in the sagittal sections of E14.5 WT and *Bax*^{tm1Sjk} mice mesencephalon. **e** FACS plots showing the gating strategy to isolate CD11b^{hi}CD45^{lo} microglia (black ellipses) from E14.5 mice mesencephalon regions. **f**, **g** Representative FACS plots (**f**) and graph (**g**) of CD11b^{hi}CD45^{lo} microglia (black ellipses) in E14.5 WT and *Bax*^{tm1Sjk} mice mesencephalon regions (WT and *Bax*^{tm1Sjk}, *n* = 4 mice). Each symbol represents one mouse. **h**, **i** Immunofluorescence of F4/80 (red), HuC/HuD (green), DAPI (blue), p-CaMKII (magenta; **h**) and p-CREB (magenta; **i**) in the sagittal sections of E14.5 mice mesencephalon region. The white arrows indicate the F4/80^{+} cells. **j** qPCR result of the *Hes1* transcriptional level in the sorted CD11b^{hi}CD45^{lo} cells from E14.5 mice mesencephalon. The data are from three independent experiments. Each dot represents an independent experiment. **k** *t*SNE plot of 17,879 CD45^{lo}Cx3cr1^{hi}CD11b^{hi} cells from E14.5 mice brains. The dots color represents different clusters. **l** Dot plot of various gene expressions in the 13 clusters in **k**. The dots color key (grey, low expression; red, high expression) indicates the average expression levels. The dots size represents the percentage of cells expressing the genes among each cluster. **m** Violin plots of the expression levels of the genes in Notch signalling pathway. Error bars, mean ± SEM. **P* < 0.05; ****P* < 0.001; *****P* < 0.0001; Unpaired two-tailed Student's t test. Source data are provided as a Source Data file.

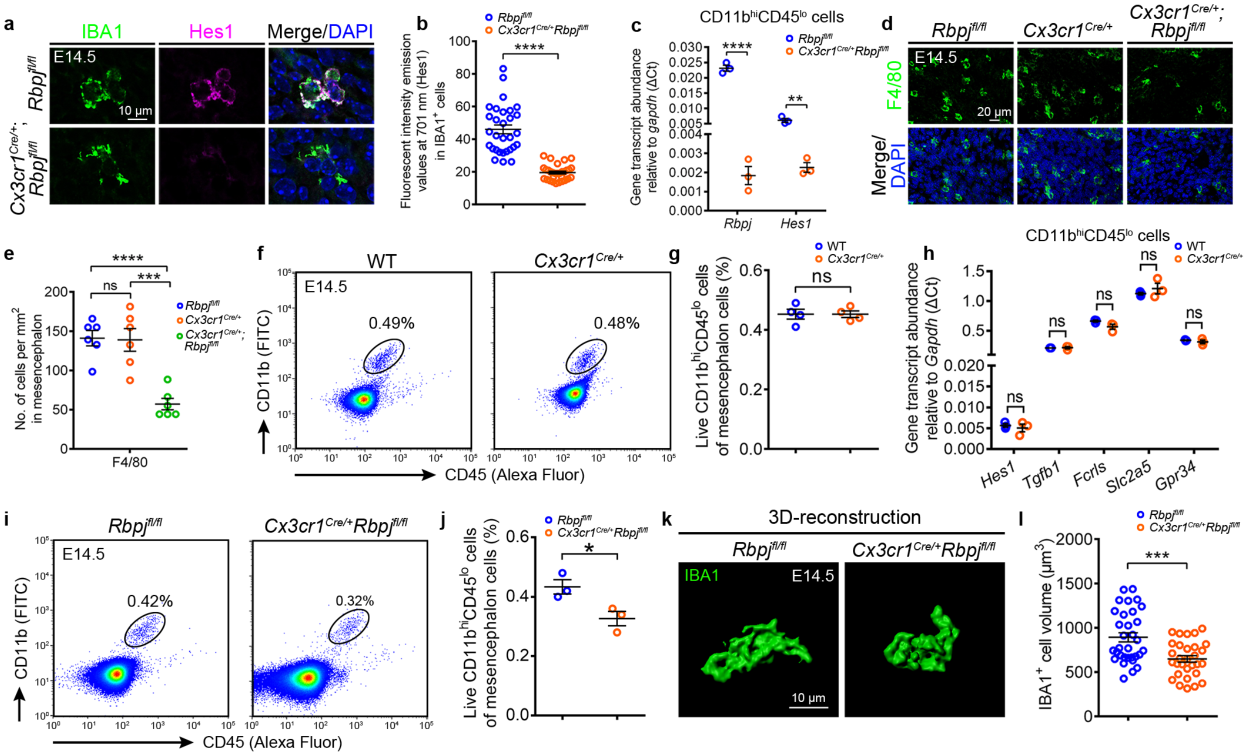

Supplementary Figure 9. The Notch signalling in microglial development is conserved in mice. a, **b** Representative immunofluorescence (**a**) and fluorescent intensity emission values (**b**) of Hes1 in the IBA$^+$ cells in the sagittal sections of E14.5 *Rbpj*$^{fl/fl}$ and *Cx3cr1*$^{cre/+}$*Rbpj*$^{fl/fl}$ mice (*n* = 3 mice in each group). Each dot in (**b**) denotes one cell. Ten cells per mouse were quantified. **c** qPCR results of *Rbpj* and *Hes1* transcriptional levels in the sorted CD11b^{hi}CD45^{lo} cells from E14.5 *Rbpj*$^{fl/fl}$ and *Cx3cr1*$^{cre/+}$*Rbpj*$^{fl/fl}$ mice mesencephalon region. The data are from three independent experiments. Each dot represents an independent experiment. **d**, **e** Immunofluorescence (**d**) and quantified cell density (**e**) of F4/80$^+$ cells in the sagittal sections of E14.5 *Rbpj*$^{fl/fl}$, *Cx3cr1*$^{cre/+}$ and *Cx3cr1*$^{cre/+}$*Rbpj*$^{fl/fl}$ mice (*n* = 6 mice in each group). Each symbol represents one mouse. Three slices per mouse were quantified. **f**, **g** Representative FACS plots (**f**) and graph (**g**) of CD11b^{hi}CD45^{lo} microglia (black ellipses) in E14.5 WT mice and *Cx3cr1*$^{cre/+}$ mice (WT and *Bax*^{tm1Sjk}, *n* = 4 mice in each group). Each symbol represents one mouse. **h** qPCR results of *Hes1*, *Tgfb1*, *Fcrls*, *Slc2a5*, and *Gpr34* transcriptional levels in the sorted CD11b^{hi}CD45^{lo} cells from E14.5 WT and *Cx3cr1*$^{cre/+}$ mice mesencephalon region. The data are from three independent experiments. Each dot represents an independent experiment. **i**, **j** Representative FACS plots (**i**) and graph (**j**) of CD11b^{hi}CD45^{lo} microglia (black ellipses) in E14.5 *Rbpj*$^{fl/fl}$ and *Cx3cr1*$^{cre/+}$*Rbpj*$^{fl/fl}$ mice (*n* = 3 mice in each group). Each symbol represents one mouse. **k**, **l** Representative 3D reconstructions of confocal z-stacks (**k**) and volume (**l**) of IBA1$^+$ cells in the sagittal sections of E14.5 *Rbpj*$^{fl/fl}$ and *Cx3cr1*$^{cre/+}$ *Rbpj*$^{fl/fl}$ mice (*n* = 3 mice in each group). Each dot in (**l**) denotes one cell. Ten cells per mouse were quantified. Error bars, mean ± SEM. **P* < 0.05; ***P* < 0.01; ****P* < 0.001; *****P* < 0.0001; ns, no significant, Unpaired two-tailed Student's t test. Source data are provided as a Source Data file.

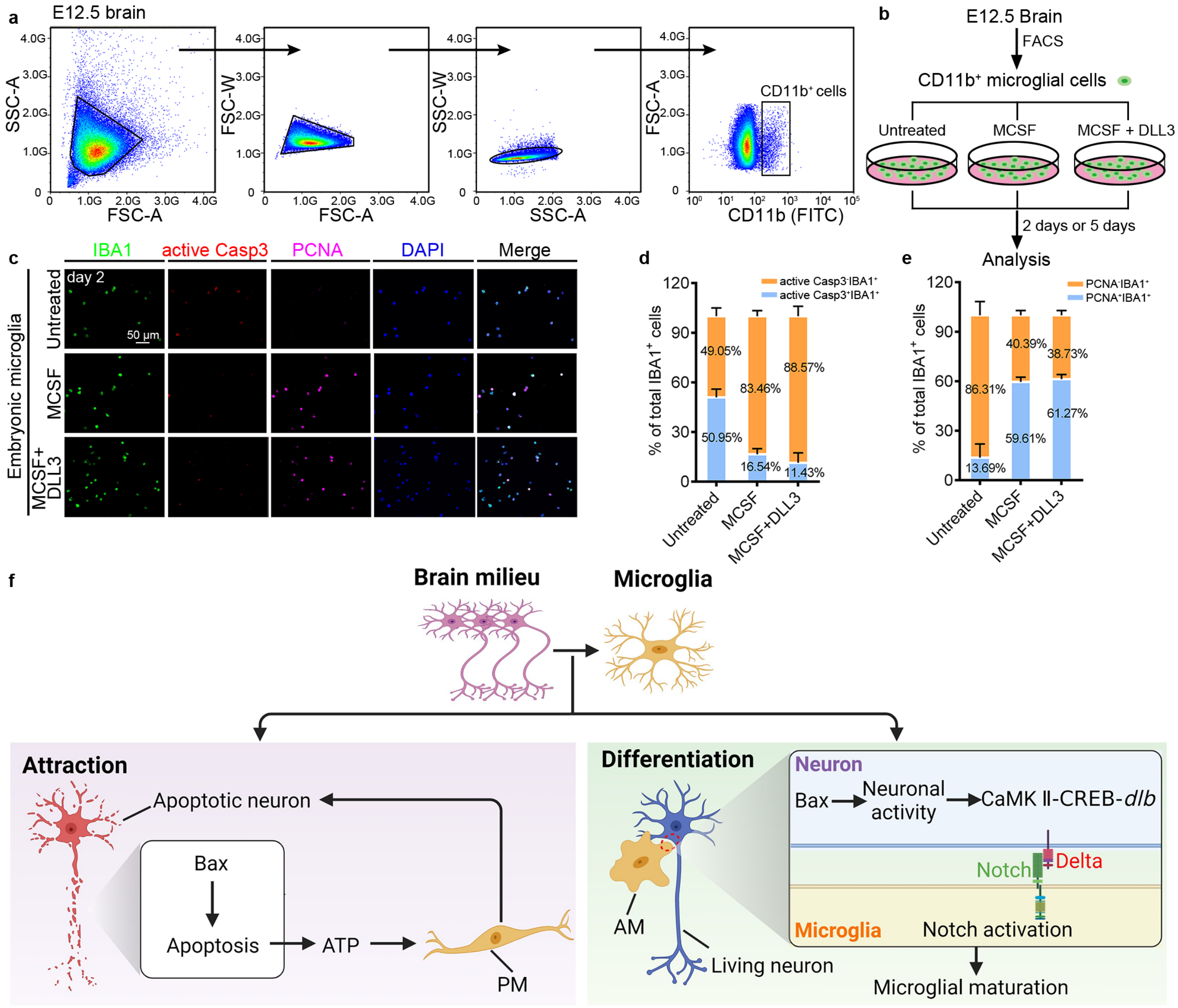

Supplementary Figure 10. Notch activity promotes microglial maturation *in vitro*. a FACS plots showing the gating strategy used to isolate CD11b$^+$ cells (black square) from the embryonic mice brains. **b** The flow chart of *in vitro* culturing isolated embryonic primary microglia, created with photoshop CS6. **c** Immunofluorescence of IBA1 and active Casp3 in the embryonic microglia furnished with MCSF or additional DLL3 *in vitro* for 2 days. **d** The percentage of active Casp3$^-$IBA1$^+$ or active Casp3$^+$IBA1$^+$ cells in **c**. The number in each histogram indicates the average percentage. Data are pooled from three independent experiments. **e** The percentage of PCNA$^-$IBA1$^+$ or PCNA$^+$IBA1$^+$ cells in **c**. The number in each histogram indicates the average percentage. Data are pooled from three independent experiments. **f** Working model, created with BioRender.com. Pre-microglia are shuttling. They move and dwell more time at where ATP or eat-me signals locate, which is given by Bax regulated apoptotic neurons. While the Notch signals, triggered by the Delta subunit from the surrounding neurons, are activated to promote the maturation of pre-microglia. Bax regulates delta transcripts via neuronal activities mediated CaMK II-CREB axis. Error bars, mean ± SEM. Source data are provided as a Source Data file.

Supplementary Table 1. The primer information.

Name	Forward (5'-3')	Reverse (5'-3')
bax^{cq55} genotype	CTCTCATATATCGTGCTGA	GGTGAAAACCTATGCAAGGC
bax$^{\Delta 1/\Delta 1}$ genotype	TCATCTATGCAGGCTCAGTG	WT: TAGGACATCGTCCAGCTTAT mut: TAGGACATCGTCCAGCTTAG
bax$^{\Delta 58/\Delta 58}$ genotype	TCATCTATGCAGGCTCAGTG	WT: TAGGACATCGTCCAGCTTAT mut: GTCATGACTTACCCATCCCT
Cx3cr^{Cre} genotype	control: CCTCAGTGTGACGGAGACAG Cre: GACATTTGCCTTGCTGGAC	GCAGGGAAATCTGATGCAAG
Rbpj$^{flox/flox}$ genotype	GTTCTTAACCTGTTGGTCGGAACC	control: GCTTGAGGCTTGATGTTCTGTATTGC flox: ACCGGTGGATGTGGAATGTGT
Bax^{tm1sjk} genotype	WT: GAGCTGATCAGAACCATCATG mut: CCGCTTCCATTGCTCAGCGG	GTTGACCAGAGTGGCGTAGG
dlb$^{-/-}$ genotype	WT: GGAGAGTGCAAGTGTCGTCT mut: TGCAAGTGTCACCGAAATAG	CATTAATCTCCAGCTCACAG
Z-*bax* cDNA	GAAGCGTCTTCTTCAGTTCG	GAACCATCTACATCTGGCTG
M-*bax* cDNA	ATGGACGGGTCCGGGGAGCA	TCAGCCCATCTTCTTCCAGA
H-*bax* cDNA	ATGGACGGGTCCGGGGAGCA	TCAGCCCATCTTCTTCCAGA
creb1a cDNA	TCCGCCTACCTCTCGGATCT	AGTCTCTGTCTCTTGAGGCA
dlb cDNA	GATAAAGTGAGCGAGATGGC	TCTCATCCCTGCATGCGAGT
NICD1a cDNA	GGTGGTCGTCTCCAGGAAG	CTACTTGAAAGCCTCTGGAAT
DN-MAML-FLAG	TCCCCCCGGGATGGTGCCGCGGCATAGTGC	CCATCGATCTTGTCGTCATCGTCTTTGT
DenNTR	CCATGAACACTCCTGGAATC	AATCAGGAGAATCTGAGAGG
pry12 probe	TTACTTCACCCAGCAGGACT	TGTGTGTCCGTGTTTTGTCC
p2ry6 probe	CCCTGAATGTTTCGACCATG	GAGGTGATTTTGAGCAGCAG
baxa-qPCR	CGCAGCTGCAAAGCATGTTA	ACAAGGCGACAGGCAAAGTA
gapdh-qPCR	ACAGTTGTAAGCAATGCCTC	GATGATGTTCTGACTGGCAC

cd36-qPCR	TCCATAAGGAAACAGTGCTG	CAGCACAAAGGACACAGTGT
gpnmb-qPCR	TTTCAACGTCCAGCTCCACG	AGGAAGAGTTGGTATTGCTG
scarb2a-qPCR	TTACGTGAGGGACATGGAAG	ACAACAATGGGTGCACCTTG
dlb-qPCR	TGCATGGGACATGTTCACAG	TCTCCAGCTCACAGTTTGTG
her4.1-qPCR	GGAGAACTGAACACAAGACAC	TGCTGTTGATTCGCTCTCG
Rbpj-qPCR	CTCACTAGGGAAGCTATGCG	GACTCTTGTTCAGAACATCC
Gapdh-qPCR	TGCACCACCAACTGCTTAG	GGATGCAGGGATGATGTTC
Dab2-qPCR	GATGATGTGCCTGATGCTC	ACGACTAATGGTTCAGCCTG
Gpr34-qPCR	GTGCCAAATGTCACTAGCTG	GAGTAGAAGGTCTGCAACAG
Hes1-qPCR	TTCCAAGCTAGAGAAGGCAG	TAGGTCATGGCGTTGATCTG
Mcm5-qPCR	CAGACATGATGTCACACCTG	CACACACTTGCACTTGTCAG
Sall1-qPCR	ACTAGCTGCCTTAGAGGAAC	TTCTGAAAGGTCGCTGCAGT
Fcrls-qPCR	TGTCCAAGAGCTGTTTCCAG	CAGTCATTGCTTCACACCAG
Slc2a5-qPCR	CTCAGAGTTCATGCAGCAGT	ACCAAGGTTCCAACCATGAG
Tmem119-qPCR	CCAGTACGTGATGCTCATCG	ATGTCAGCCTGGAGCTGATG
P2ry12-qPCR	GCGTCAGAGACTACAAGATC	GTAGATCAGAGATGACCGTG
Pros1-qPCR	AGCCATCTCAGACCAATGTG	TGTCACAAATCTGGCTGCAG
Tgfbr1-qPCR	CTGGATCAGGTTTACCACTG	CCATGAACGCTCTTCTCTAG
C1qa-qPCR	ACAGTGGCTGAAGATGTCTG	TCATTGGGTTCTGCCGAATG
Hexb-qPCR	ACTAGCTGCCTTAGAGGAAC	TCTCATCCAGCTCTACACAG
dlb-ChIP-site1	CACATGCAGCAGCAGATCAA	GTTCGTCTTGATGAATGGAC
dlb-ChIP-site2	GTATGTGCTTATTTGATGCT	ATGCATCCTCAAATCCTGAG
dlb-ChIP-NC	ATTGGAGAACCCATCTGCTC	TTGATCTGCTGCTGCATGTG
MO-*creb1a*	5'-CTAGATAGCCCGACACAGACTCGCC-3'	
MO-*dlb*	5'-CGCCATCTCGCTCACTTTATCCTAA-3'	
MO-*notch1a*	5'-GTAGTGTTAAACTGTTACCTTGTGC-3'	
